# O.6.3-8 The challenge of participatory projects for physical activity promotion: what to scale and how to scale?

**DOI:** 10.1093/eurpub/ckad133.288

**Published:** 2023-09-11

**Authors:** Raluca Sommer, Maike Till, Alexandra Sauter, Karim Abu-Omar, Peter Gelius

**Affiliations:** Friedrich-Alexander-Universität Erlangen-Nürnberg, Germany; Friedrich-Alexander-Universität Erlangen-Nürnberg, Germany; University of Regensburg, Germany; raluca.sommer@fau.de; Friedrich-Alexander-Universität Erlangen-Nürnberg, Germany; Friedrich-Alexander-Universität Erlangen-Nürnberg, Germany

## Abstract

**Purpose:**

Effective pilot interventions for physical activity (PA) promotion should be scaled up, i.e. transferred to broader contexts while permanently integrating them into policies or systems (WHO, 2010). This study explores the scale-up strategies of four German projects that developed actions for PA promotion based on a participatory approach (Gelius et al., 2021), highlighting the challenges encountered while transferring the participatory approach to other contexts and reaching other population groups.

**Methods:**

We conducted a qualitative study based on document analysis and 15 semi-structured focus group interviews with each of the projects’ research teams. The ExpandNet framework (WHO, 2010) was used to retrospectively analyze the scale-up strategies as well as key elements (i.e., innovation, user organization, environment) utilized in the projects.

**Results:**

The projects succeeded in transferring effective pilot interventions to a total of 16 new sites (“horizontal” scale-up). At some sites, the process ran smoothly, while at others, the employed scale-up strategies differed from the initial plan. As a result, some projects scaled the participatory component, while others the specific actions previously developed in the pilot project. Also, in some projects, political support and other favorable circumstances allowed for institutionalizing PA promotion actions on a higher organizational level (“vertical” scale-up).

**Conclusions:**

When participatory approaches are used as mechanisms to co-create an agenda for PA promotion jointly with researchers, population groups, and relevant stakeholders, it seems that multiple strategies may be employed for scale-up. Participatory projects can be scaled-up horizontally or vertically, i.e. by focusing either on the participatory approach as a whole or on specific actions developed using the participatory approach in a previous pilot project. There is a need to develop novel theoretical concepts of scalability that capture the diversity of approaches observed in our study.

**Note:**

This study was conducted within the Capital4Health, a consortium funded by the German Federal Ministry of Education and Research [01EL1821A-F].

